# Correspondence on “Marine Scrubbers vs Low-Sulfur Fuels: A Comprehensive Well-To-Wake Life Cycle Assessment Supported by Measurements Aboard an Ocean-Going Vessel”

**DOI:** 10.1021/acs.est.5c15904

**Published:** 2026-07-30

**Authors:** Fayas Malik Kanchiralla, Anna Lunde Hermansson, Roland Pfeiffer, Selma Brynolf, Erik Ytreberg, Ida-Maja Hassellöv

**Affiliations:** † 11248 Chalmers University of Technology, Chalmersplatsen 4, 412 96 Gothenburg, Sweden; ‡ IVL Swedish Environmental Research Institute, 400 14 Gothenburg, Sweden

Regarding the extensive work by Stathatou et al.,^1^ which includes onboard measurements in well-to-wake (WtW) LCA, it is important to examine the selection of goal and scope definition, inventory analysis, and the interpretation of results in the LCA method used. With the limitations applied in the study, the strongly stated conclusion raises the concern of downplaying potential risks to a degree that undermines the ongoing global discussion within the IMO on the use of scrubbers.

## Interpretation of Results

### Critical Impact Categories Are Omitted

Stathatou et al.^1^ state that, “Considering all the LCA impact categories, it can be concluded ... HFO with a scrubber can be considered equal to the use of MGO, while outperforming VLSFO in several impact categories ...”. This generalization conflicts with their own findings in Figure 5, where tank-to-wake (TtW) impacts for marine ecotoxicity and marine eutrophication (the impact categories directly relevant to marine ecosystems) are several times higher than those from VLSFO and MGO. These critical aspects are dismissed with reference to large uncertainties and the lack of established characterization factors. Dismissing those impact categories out of hand due to uncertainty in the data, despite the fact that the results indicate large negative effects in those impact categories, raises concerns regarding the interpretation of the results. There are uncertainties for all other impact categories, but the impact categories (ecotoxicity and eutrophication) with the largest differences are specifically dismissed without any details of what characterization factors are lacking that affect results.

### Discriminating Ecotoxicity and Marine Eutrophication

First, consider the Statement by Stathatou et al.:^1^ “Assuming a conservative initial dilution factor of 10, marine eutrophication and ecotoxicity TtW impacts get lower than the WtT ones ...”. This approach disregards the basis on which characterization factors are derived. If the assumption were applicable, an analogue could be made for all of the other impact categories where, e.g., CO_2_ from a single ship is diluted in the atmosphere after emission. Second, the result in Figure 5 does not support the following statement in ref 1: “... relevant results ... cannot be used to conclude that HFO with scrubber has greater eutrophication or ecotoxicity impacts compared to low-S fuels.” In fact, the marine ecotoxicological impact is likely greater, as many substances in scrubber effluents (e.g., alkylated polycyclic aromatic hydrocarbons with higher toxicity potential than their parent derivatives^4^) are, critically, not included in ReCiPe.^2,3^

## Methodological Inconsistencies

### Selection of the Functional Unit

The functional unit needs to be a reference flow, but megajoule fuel input (MJin) is rather an inflow to the ship and hence not specifically a reference flow as it is not related to function, i.e., transport work. The choice of MJ_in_ will specifically omit the energy penalty and efficiency improvement (both technical and operational) of the ship’s system. Thus, although the study claims that the energy penalty is included, by using MJin as the functional unit, it ignores the energy penalties associated with scrubber operation. To clarify, scrubber systems require 80–100 kW of electrical power, which is approximately a 2% energy penalty on average ship operations, implying that the corresponding transport work accomplished with 1 MJ of MGO input would require 1.02 MJ equivalent of HFO when operating with scrubbers. Selecting MJ of fuel as the functional unit discards these energy penalties (as the calculation cancels out additional energy use and related emissions by reconverting to MJin).^5^ The IMO LCA guidelines use MJ as a functional unit, but this should not be used as an argument for the scientific accuracy. The purpose of the IMO LCA guidelines is different as it is developed to harmonize data to be used in IMO climate policy frameworks and not to compare the environmental impacts of ships using different propulsion systems. Rather, applying MJ as functional units will result in misrepresentation of the absolute environmental impact of HFO-scrubber systems.

### Analyses of Marine Ecotoxicity and Eutrophication

Information about the specific substances included in the analysis of marine ecotoxicity and eutrophication and how concentrations (presumably μg/L, although written as μm/L) are transformed from grams of 1,4-DCB equivalents per liter (g 1,4-DCBEq/L) to g 1,4-DCBEq/MJ is lacking. In the main text, the marine ecotoxicity is reported as 0.07 g 1,4-DCB_Eq_/MJ_in_ (over the scrubber’s 20-year lifetime), which is then erroneously converted to 0.07 1,4-DCB_Eq_/m^3^ of discharged scrubber water (Note 14 of the Supporting Information (SI)). Instead, translation to g 1,4-DCB_Eq_/MJ should account for the scrubber discharge flow rate (68 m^3^/MWh indicated in the main text and Figure S17, Table S5, and Figure S19) and kWh transformed to MJ (7.8 MJ_in_ for 1 kWh engine out), resulting in 0.07 g 1,4-DCB_Eq_/MJ = 8.1 g 1,4-DCB_Eq_/m^3^. This implies 35 times higher marine ecotoxicity than previously reported (comparison to 0.23 g 1,4-DCB_Eq_/m^3^ of scrubber discharge^3^ in Note 14 of the SI) and is equivalent to increasing the average marine ecotoxicity damage cost in the Baltic Sea in 2018 from €32 million to €1100 million, only from discharges of open loop scrubber water.

### Different Engine Conditions

Table S5 shows that the engine conditions are different for each fuel scenario considered, which will result in uncertainty in the TtW calculations as they are recalculated to MJ_in_ based on an average engine efficiency of ∼0.46 (Note 3 of the SI).

### Selection of Background Inventory Data

The selection of background data for the WtW assessment appears to be automotive diesel, not MGO, calling into question the relevance of the analysis. Automotive diesel represents fuel with 10 ppm sulfur (EuroVI), compared to 1000 ppm sulfur in MGO, which affects the refining processes and associated emissions. The Ecoinvent database for refining is based on the work of Jungbluth et al.,^6^ which reports a WtT carbon intensity of 18.6 kg of CO_2_ equivalents for low-sulfur fuel oil (0.1% S) versus 17.4 kg of CO_2_ equivalents for HFO, an ∼6% difference. However, Figure 6 shows a difference of more than 15% between HFO and MGO, suggesting inappropriate background data.

In summary, the issues in the methodological choices used in the LCA and the selective interpretation and/or dismissal of other results call into question the analysis presented in ref 1. In light of the errors and analysis gaps discussed here, the conclusions in ref 1 regarding the environmental performance of marine scrubber technology deserve a closer look.
